# Public Cord Blood Banks as a source of starting material for clinical grade HLA-homozygous induced pluripotent stem cells

**DOI:** 10.1186/s13287-022-02961-6

**Published:** 2022-08-12

**Authors:** Belén Álvarez-Palomo, Anna Veiga, Angel Raya, Margarita Codinach, Silvia Torrents, Laura Ponce Verdugo, Clara Rodriguez-Aierbe, Leopoldo Cuellar, Raquel Alenda, Cristina Arbona, Dolores Hernández-Maraver, Cristina Fusté, Sergi Querol

**Affiliations:** 1 Cell Therapy Service, Banc de Sang i Teixits, Edifici Dr. Frederic Duran i Jordà, Passeig de Taulat, 106-116, 08005 Barcelona, Spain; 2 Transfusional Medicine Group, Vall d’Hebron Research Institute, Autonomous University of Barcelona (UAB), Barcelona, Spain; 3 Programa de Medicina Regenerativa, Institut d’Investigació Biomèdica de Bellvitge. IDIBELL, Hospital Duran i Reynals, Gran Via de L’Hospitalet, 199-203, 08908 L’Hospitalet de Llobregat, Barcelona, Spain; 4 Musculoskeletal Tissue Engineering Group, Vall d’Hebron Research Institute, Autonomous University of Barcelona (UAB), Barcelona, Spain; 5 Centro de Transfusión, Tejidos y Células de Málaga, Avda. Doctor Gálvez Ginachero s/n, 29009 Malaga, Spain; 6 Basque Center for Blood Transfusion and Human Tissues, Osakidetza, Barrio Labeaga 46A, 48960 Galdakao, Spain; 7 Cell Therapy, Stem Cells and Tissues Group, Biocruces Bizkaia Health Research Institute, 48903 Barakaldo, Spain; 8 Axencia Galega de Sangue, Órganos e Tecidos, Rúa Xoaquín Díaz de Rábago 2, 15705 Santiago, Spain; 9 Centro de Transfusión de la Comunidad de Madrid, Avda. de la Democracia, s/n, 28032 Madrid, Spain; 10 Centro de Transfusión de la Comunidad Valenciana, Av. del Cid, 65-acc, 46014 Valencia, Spain; 11 Fundacion para el Fomento de la Investigación Sanitaria de la Comuitat Valenciana, Avda. de Catalunya, 21, 46020 Valencia, Spain; 12 BM and CB Transplant Program, Organización Nacional de Trasplantes, Madrid, Spain; 13 REDMO/Fundació i Institut de Recerca Josep Carreras, C/Muntaner, 383 2n, 08021 Barcelona, Spain

**Keywords:** Hematopoietic progenitor cells, Cord blood, Cord blood banks, Induced pluripotent stem cells, HLA matching, GMP manufacturing

## Abstract

**Background:**

The increasing number of clinical trials for induced pluripotent stem cell (iPSC)-derived cell therapy products makes the production on clinical grade iPSC more and more relevant and necessary. Cord blood banks are an ideal source of young, HLA-typed and virus screened starting material to produce HLA-homozygous iPSC lines for wide immune-compatibility allogenic cell therapy approaches. The production of such clinical grade iPSC lines (haplolines) involves particular attention to all steps since donor informed consent, cell procurement and a GMP-compliant cell isolation process.

**Methods:**

Homozygous cord blood units were identified and quality verified before recontacting donors for informed consent. CD34+ cells were purified from the mononuclear fraction isolated in a cell processor, by magnetic microbeads labelling and separation columns.

**Results:**

We obtained a median recovery of 20.0% of the collected pre-freezing CD34+, with a final product median viability of 99.1% and median purity of 83.5% of the post-thawed purified CD34+ population.

**Conclusions:**

Here we describe our own experience, from unit selection and donor reconsenting, in generating a CD34+ cell product as a starting material to produce HLA-homozygous iPSC following a cost-effective and clinical grade-compliant procedure. These CD34+ cells are the basis for the Spanish bank of haplolines envisioned to serve as a source of cell products for clinical research and therapy.

**Supplementary Information:**

The online version contains supplementary material available at 10.1186/s13287-022-02961-6.

## Background

Induced pluripotent stem cells (iPSC) can be generated in the laboratory from donor adult somatic cells by the reprogramming process, displaying similar characteristics to embryonic stem cells and free of the ethical concerns related to embryo use [1]. IPSC can be expanded unlimitedly and they can be differentiated into any cell type in the body; moreover, the genetic background of the donor can be chosen. These features make iPSC exceptional candidates as starting material for cell therapies [2]. IPSC-derived cells are being tested at the moment in clinical trials for regenerative medicine to treat diseases such as age-related macular degeneration [3] or Parkinson’s disease [4], and for immunotherapies for conditions such as cancer [5] or graft-versus-host disease [6].

Although the production of therapeutic cells from the patient’s own iPSC would provide no graft rejection due to histocompatibility issues, the high cost and the long duration of iPSC production plus differentiation to the therapeutic cell aroused considerations over the practicality of an autologous use of iPSC. Several approaches for the allogenic use of iPSC have been proposed, like genome editing to eliminate HLA expression [7] or HLA-matching, similarly to what is done for organ or bone marrow transplantation [8]. It was proposed that creating iPSC lines from HLA homozygous donors would provide a match for a wide percentage of the population with a relatively small number of iPSC lines [9]. This approach has shown promising results in primates [10]. Since HLA haplotypes differ in each geographical area, several studies have identified the most common haplotypes that would serve a national or regional population and calculated the number of HLA homozygous iPSC lines (haplolines) to cover a significant percentage [9, 11, 12]. Cord blood banks provide an excellent source of starting material for the production of iPSC: the samples are banked in great numbers, they are already HLA-typed and screened for the presence of virus and pathologies [13]. Moreover, CB cells are young cells with negligible risk of accumulated genetic and epigenetic insults and easy to reprogram. Several countries have set up initiatives to create their own banks of haplolines (haplobanks) using banked CB as a source of starting cells, with Japan and Korea reporting already the first haplolines [14, 15].

In order to use iPSCs in cell therapy, haplolines must be produced to clinical grade, complying with GMP standards throughout manufacturing [16]. Several international efforts have been put in place to create a set of standards for critical quality attributes of iPSC for clinical applications, seeking the international harmonization to allow for haplolines exchange among haplobanks and a better coverage of the population [17, 18].

Several crucial aspects have to be considered in the production of clinical grade iPSC, starting with the procurement of the starting cells. Banked CB provides a source of cells that have been already procured following clinically regulated standards such as Jacie [19] or Netcord [20], that assure the compliance with therapeutic use standards; however, their use for reprogramming requires signing a new informed consent explaining the novel applications of the donation.

The most common strategies to obtain haplolines from blood samples consists in isolating the mononuclear fraction or the CD34+ hematopoietic progenitor population [21, 22].

In this article, we report our experience to obtain CD34+ from homozygous donors to make iPSC haplolines following a procedure that complies with regulatory standards for ATMP starting materials and that can be adopted by other CBB with an interest in creating iPSC haplobanks. The process was performed within the Spanish haplobank project (IPS-PANIA) on nine CB units corresponding to HLA haplotypes estimated to provide the widest possible coverage of the Spanish population [12].

## Materials and methods

### Safety control of CBU accepted in the bank

All the CBU candidate for being included in a CBB are tested for microbial sterility by colorimetric culture assay and for the presence of antibodies against common blood borne viruses: hepatitis B (HBV), VIH 1/2, hepatitis C (HCV), HTLV-I/II, cytomegalovirus and Epstein Barr and for the presence of VHB DNA and HVC and VIH-1 RNA. The presence of antibodies against parasites Toxoplasma gondii, Trypanosoma cruzi and Treponema pallidum are also assessed.

### Segment verification

High-resolution typing was performed by Sanger sequencing in an ABI PRISM 3130xl Genetic Analyzer (ThermoFisher) and/or next generation sequencing (NGS) in a MiSeq platform (Illumina) or in Ion GeneStudio S5 System (ThermoFisher) for HLA-A, -B, -C, -DRB1, -DRB3/4/5, -DQB1 and -DPB1 genes. CD45+ and CD34+ cell content was assessed by flow cytometry following ISHAGE protocol and viability was calculated by dye exclusion with 7AAD. Colony formation assays to calculate the expected clonogenic efficiency (E-clone) was done in StemMACS HSC-CFU methyl-cellulose medium (Miltenyi) and cultivation for 14 days in a CO_2_ cell 37 °C, 5.5% CO2, 90% HR incubator.

### Thawing of the CBU and mononuclear fraction (MNF) purification

The CB bags were thawed in a water bath at 37 °C and slowly equilibrated with a 1:1 volume of equilibration buffer containing PBS with no Ca2+ or Mg2+, 5% p/v human serum albumin (Griffols), 5 mM MgCl_2_ and 200U/mL of DNAse I (Pulmozyme, Roche) in a closed system and allowed to equilibrate at 4 °C for 5 min. The mononuclear (MN) fraction was purified in a closed system using ficoll (Histopaque-1077, Sigma-Aldrich) in a Sepax-2 cell processor (Biospace) with the NeatCell program and using the CS900.2 kit. PBS with no Ca2+ or Mg2+, 2% p/v human serum albumin (Griffols), 2.5 mM MgCl_2_ and 20U/mL of DNAse I (Pulmozyme, Roche) was used as washing buffer. The obtained MNF was diluted 1:1 with washing buffer then centrifuged for 15 min at 300 g. The supernatant was discharged and the pellet resuspended at a concentration of 2 × 10^6^ MN cells/mL in washing buffer.

### CD34+ selection

The cell suspension was blocked for 5 min at 4 °C with 100 ul/10^8^ cells of Planngamma 100 mg/mL (Griffols). CliniMACS CD34 reagent (Miltenyi) immunomagnetics beads were added at 100 ul/10^8^ cells and incubated for 30 min at room temperature, mixing every 5 min. Subsequently the cells were washed in 50 mL of washing buffer and centrifuged 15 min at 300 g. The labeled cells were then passed through a clinical grade separation mini column (MACS ART MS columns Miltenyi) located in a fixed magnetic field. For each 10^8^ cells two consecutive columns were used. CD45+ and CD34+ content and viability were assessed right after purification and after o/n incubation by flow cytometry using anti-CD45 (clone J33, Beckman), anti-CD34 (clone 581, Beckman) and 7AAD (Beckman) for viability.

### CD34+ expansion

CD34+ selected cells were resuspended and cultivated in Stempro34 (Gibco) supplemented with IL6 10 ng/mL, IL3 10 ng/mL, TPO 10 ng/mL, SCF 50 ng/mL and Flt3l 50 ng/mL. All cytokines were human recombinant GMP grade (PeproTech).

### Sterility and mycoplasma testing

Sterility was assessed by BactAlert IFA and iFN plus bottles (Biomerieux) and the presence of mycoplasma by MycoAlert™ Detection Kit (Lonza).

## Results

**Fig. 1:**
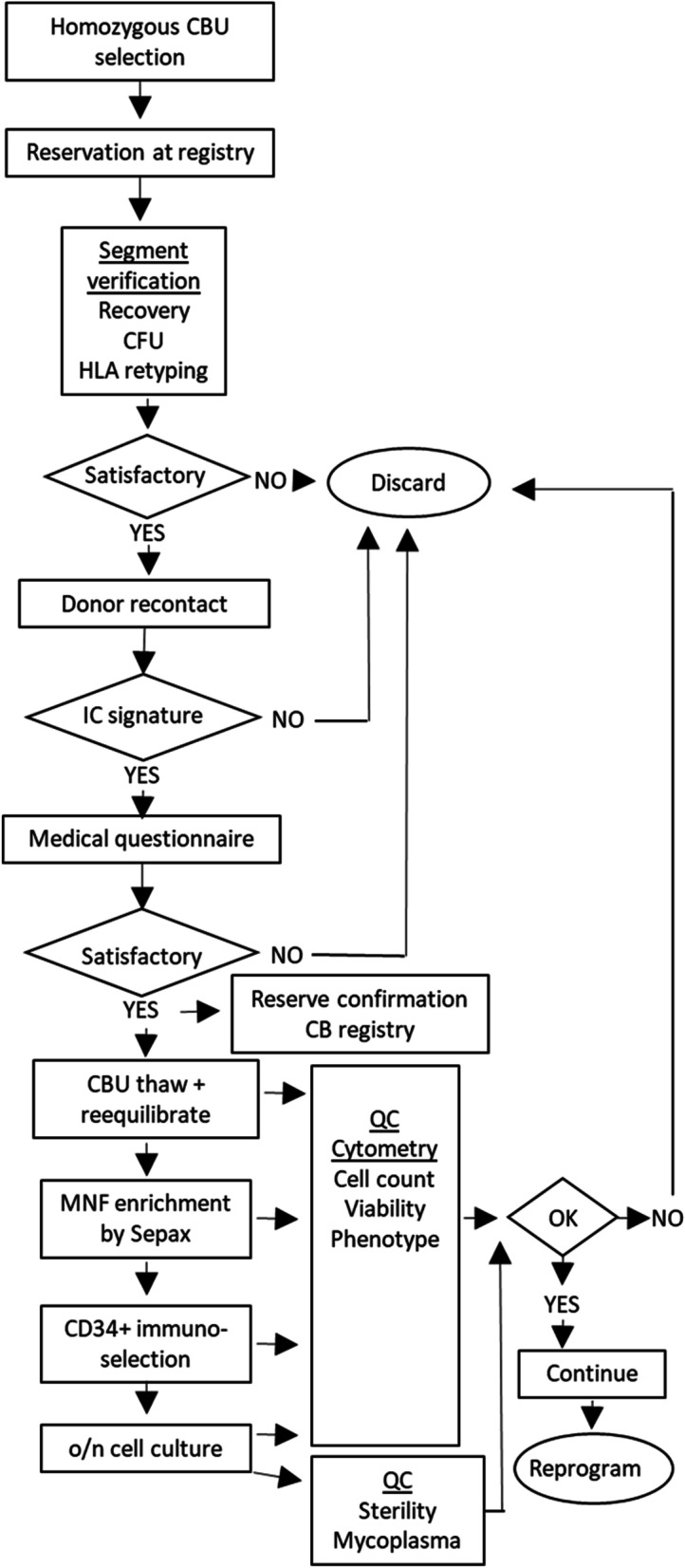
Process diagram for homozygous CB units (CBU) selection and processing. IC = Informed Consent

An overview of the process we followed to obtain clinically compatible homozygous CD34+ is depicted in Fig. 1, with critical steps and go/no go points.

### Donor recontact and reconsent

All cord blood donors—the mothers of the new-borns whose umbilical cord blood is collected—recontacted had agreed in the informed consent for the original cord blood donation on the possibility of being contacted again in the future. The donors were telephoned and informed about the project and the special interest of their homozygous donated samples. Subsequently, an e-mail was sent including an introductory brochure on simple language, the Donor Information document and the Informed Consent document, and they were invited to read them carefully; both documents had been approved by the Vall d'Hebron Hospital Ethics Committee for Human Research. The donors that were interested in participating had an interview, either in person or by telephone, with a physician who knew the project in detail but was not directly involved in it. Those donors consenting on participating also answered a health questionnaire to discard genetic diseases or other incompatibilities with the donation (Additional file 1: Fig. S1.).

All the selected donors were reachable by phone and willing to listen to the proposal. An in-person interview was the first choice to meet the donor and to give them a chance to ask questions about the donation and the project, and to sign the Informed Consent document. However, due the COVID-19 pandemic, for most of the donors the interviews were performed over the phone and the Informed Consent was sent by courier to the donor's home and collected on the spot.

From the 17 donors recontacted, only three of them declined to participate and one of them was discarded due to a chromosomal abnormality found in a post-donation miscarriage.

### CBU selection criteria

The whole Spanish cord blood registry comprises 52,220 donations. From the 9419 which are genotyped in high resolution, we identified 109 homozygous units corresponding to 43 different haplotypes and we ranked the haplotypes priority according to the cumulative coverage capacity for HLA matching, as previously described [12]. The average pre-freeze content in CD34+ in the homozygous units was 5.51 × 10^6^. The screening selection criteria for the homozygous CBU are listed in Table 1.

**Table 1:** Homozygous CB units initial selection criteria

Available CBU in Spanish banks	≥ 2
CD34+ count pre-freeze [10E+06]	≥ 2
Post-thaw viability CD34+ (%)	≥ 50
E-clone (%)	> 10
ABO type	0

As an initial selection criterion, only those haplotypes represented in more than two homozygous cord blood units were considered, in order to keep always at least one unit of the haplotype available for transplantation within any of the national Cord Blood Banks. A second selection criteria was to have a pre-freeze CD34+ content of ≥ 2 × 10^6^ cells. Selected units were pre-reserved from the Spanish CB registry. Verifications on frozen segments were performed for 25 units with the goal to create seven haplolines representing the top haplotypes estimated to give the widest coverage for the Spanish population. Initially, we set as acceptance criteria having an E-clone of at least 10%, as a measure of hematopoietic progenitor potency, and a CD34+ viability after thawing of at least 50%. Since segments normally perform worse than the main bag in terms of viability upon freezing, some samples with low performance on CD34+ viability or colony formation at the verification, were still considered as candidates taking into account other considerations such as CD34+  content at collection or the quality of alternative candidate CBU for that haplotype. On the thawed segments, the median viability of CD45 + cells was 51.4% [15.9–88%] and that of CD34+ was 82% [36.8–98.7%] and the median E-clone was 24.6% [0.6–130.9%] (Table 2).

**Table 2:** Quality control data from CB units segments verifications

Haplotype	Rank cover	CBU	Freeze date	Pre-freeze		Post-thaw			SEX	ABO
				NC ×10e9	CD34+ ×10e6	CD45+ 7AAD- %	CD34+ 7AAD- %	Eclone %		
**29:01–44:03–07:01**	**1**	**1**	**04/11/2010**	**2.68**	**8.7**	**15.9**	**36.8**	**3.6**	**M**	**O POS**
		2	11/20/2009	2.41	11.8	34.8	62.4	28.0	M	O POS
		3	05/17/2013	2.19	7.9	47.0	81.0	19.6	F	O NEG
		4	10–11-2008	0.93	2.8	53.1	70.0	75.9	F	O NEG
**01:01–08:01–03:01**	**2**	**1**	**12–06-2009**	**1.63**	**17.9**	**76.8**	**89.0**	**20.4**	**F**	**O NEG**
		2	10/27/2009	2.23	6.8	36.7	49.6	11.0	F	A POS
		3	10/30/2008	0.95	8.29	88.0	90.0	7.2	F	O POS
**30:02–18:01–03:01**	**3**	**1**	**05/23/2015**	**2.10**	**7.2**		**90.4**	**15.9**	M	O POS
		2	12/20/2004	1.67	6.8	31.0	66.0	0.56	F	O POS
		3	03–04-2009	2.18	5.2	56.9	73.0	71.1	F	O NEG
**03:01–07:02–15:01**	**4**	**1**	**04/10/2007**	**1.88**	**11.4**	**77.2**	**91.0**	**46.6**	**M**	**O POS**
		2	02/25/2003	2.40	5.5	50.0	86.0	8.2	M	B POS
		3	06/16/2008	2.05	12.6	45.0	65.0	27.0	F	A POS
**02:01–44:03–07:01**	**5**	1	**03/04/2008**	**1.06**	**2.9**	**61.2**	**82.0**	**41.1**	**M**	**O POS**
		2	03/02/2011	1.17	2.3	70.0	70.0	130.9	M	A POS
02:01–07:02–15:01	6	1	05/15/2009	1.55	9.3	81.3	86.0	12.1	F	B POS
**33:01–14:02–01:02**	**7**	**1**	**02/12/1999**	**0.96**	**5.1**	**50.0**	**78.0**	**13.9**	**F**	**O NEG**
		2	03/17/2003	1.26	2.7	45.0	98.7	49.8	M	O POS
02:01–51:01–11:01	8	1	04/14/2018	2.30	2.7			85.5	F	B POS
		2	11/14/2006	1.70	6.7	51.0	91.0	21.0	F	O POS
01:01–57:01–07:01	10	1	03/21/2008	2.17	2.8	51.7	86.6	36.0	F	O NEG
		2	09/27/2001	0.89	1.2	62.0	91.0	41.2	F	A NEG
**24:02–07:02–15:02**		**1**	**02/27/2016**	**1.86**	**9.0**	**42.4**	**71.8**	**34.0**	**M**	**O POS**
**11:01–27:05–01:01**		**1**	**02/05/2015**	**1.47**	**5.2**	**35.9**	**58.3**	**34.5**	**F**	**O POS**
		2	10/14/2003	1.10	3.4	82.1	95.0	25.9	F	A POS
11:01–35:01–14:54		1	09/21/2009	1.00	4.2	86.6	94.0	18.6	F	O POS
			Median	1.63	6.1	51.4	82.0	24.6		
			Range	0.89–2.68	1.2–17.9	15.9–88	36.8–98.7	0.6–130.9		

Several units were verified for each of the haplotypes that provided higher HLA matching coverage on the Spanish population (Rank cover.). NC = nucleated cells. Lines in bold correspond to the CB units finally selected for thawing

As part of the verification process, the samples HLA was retyped by NGS to confirm homozygosity. Finally, nine units were thawed for CD34+ selection, prior reserve confirmation to the Spanish CB registry. The characteristics of the selected CBU are described in Table 3.

**Table 3:** Selected CB units characteristics: High resolution HLA typing, sex, ABO type, volume of CB on collection and days in storage from collection to thawing

CBU	Freq. haplotype	HLA-A	HLA-B	HLA-C	HLA-DRB1	HLA-DQB1	HLA-DPB1	ABO type	Sex	CB volume collected (mL)	Days in storage
1	0.0311	29:02	44:03	16:01	07:01	02:02	11:01	O+	XY	141.5	3709
2	0.0262	01:01	08:01	07:01	03:01	02:01	03:01/04:01	O+	XX	88	4459
3	0.0262	01:01	08:01	07:01	03:01	02:01	01:01/03:01	O−	XX	134	4078
4	0.0196	30:02	18:01	05:01	03:01	02:01	02:02/04:01	O+	XY	115.5	1958
5	0.0135	03:01	07:02	07:02	15:01	06:02	02:01/04:01	O+	XY	130	5035
6	0.0083	02:01	44:03	16:01	07:01	02:01	11:01/19:01	O+	XY	78	4748
7	0.0096	33:01	14:02	08:02	01:02	06:02	02:01P	O−	XX	123	8339
8	0.0052	24:02	07:02	07:02	15:01	06:02	04:01P	O+	XY	126.5	1864
9	0.0040	11:01	27:05	01:02	01:01	05:01	04:01/11:01	O+	XX	105.5	2338

### Thawing of CBU and CD34+ selection

We designed a thawing and CD34+ cell isolation process that could be consistent and that yielded a sufficient number of viable CD34+ cells for reprogramming and set the acceptance criteria for the final CD34+ purified population, represented in Table 4.

**Table 4:** Acceptance criteria for the immunoselected CD34+ purified population to continue into reprogramming process

CD34+ count [10E+05]	≥ 1
CD34+ viability (7AAD neg %)	≥ 80
CD34 purity (%)*	≥ 70
Sterility	Negative
Mycoplasma	Negative

*Purity calculated as % of viable CD34+ within the CD45+ viable population

**Fig. 2:**
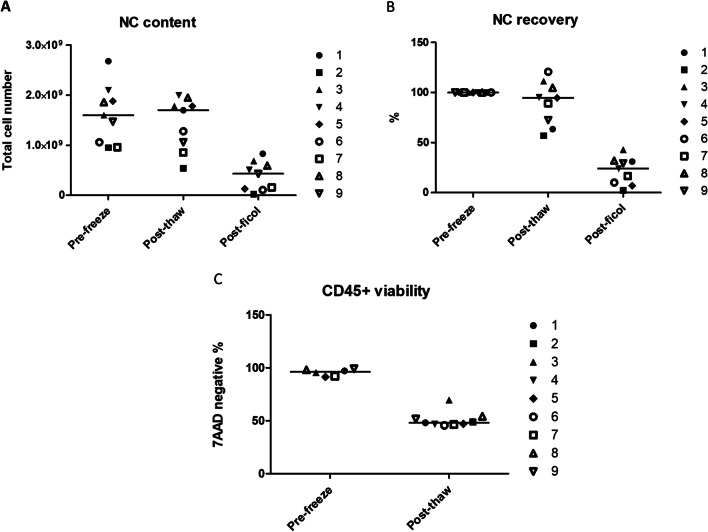
Nucleated cells (NC) recovery during the process. **A** Total number of NC measured in automatic cell counter. **B** Recovery or % vs the initially reported NC content upon collection. **C** CD45 + viability measured by dye exclusion at the cytometer, before and after freezing. Lines depict median values

The whole thawing, mononuclear fraction (MNF) enrichment and CD34+ purification process was performed in the clean room facilities—in an A grade hood within a D grade environment—and using GMP grade reagents compatible with clinical use. Each CBU was retrieved from liquid nitrogen storage and code verified before transporting it in a dry shipper to the clean room. Upon the point of MNF purification, the procedure was performed in a closed system. The CBU thawed at 37 °C in a water bath were slowly equilibrated with a 5% p/v human serum and DNAse I containing solution and a sample was retrieved for a cytometry assay. At this point the median content on nucleated cells (NC) was 1.77 × 10^9^ [5.41 × 10^8^–2 × 10^9^] cells, meaning a median recovery 94.7% [63.4–111.3], with a median viability of CD45+ of 48% [45.6–69.4%] (Fig. 2). Isolation of the MNF with the ficoll method was performed in a Sepax 2 cell processor, using the NeatCell program, directly from equilibration without prior washing. This method allowed for a closed system and consistent recovery of the MNF with ficoll. At this point, the NC content was reduced to a median 4.3 × 10^8^ [2 × 10^8^–8.3 × 10^8^] which correspond to a 25.3% [3.7–48.9%] of recovery (Fig. 2).

**Fig. 3:**
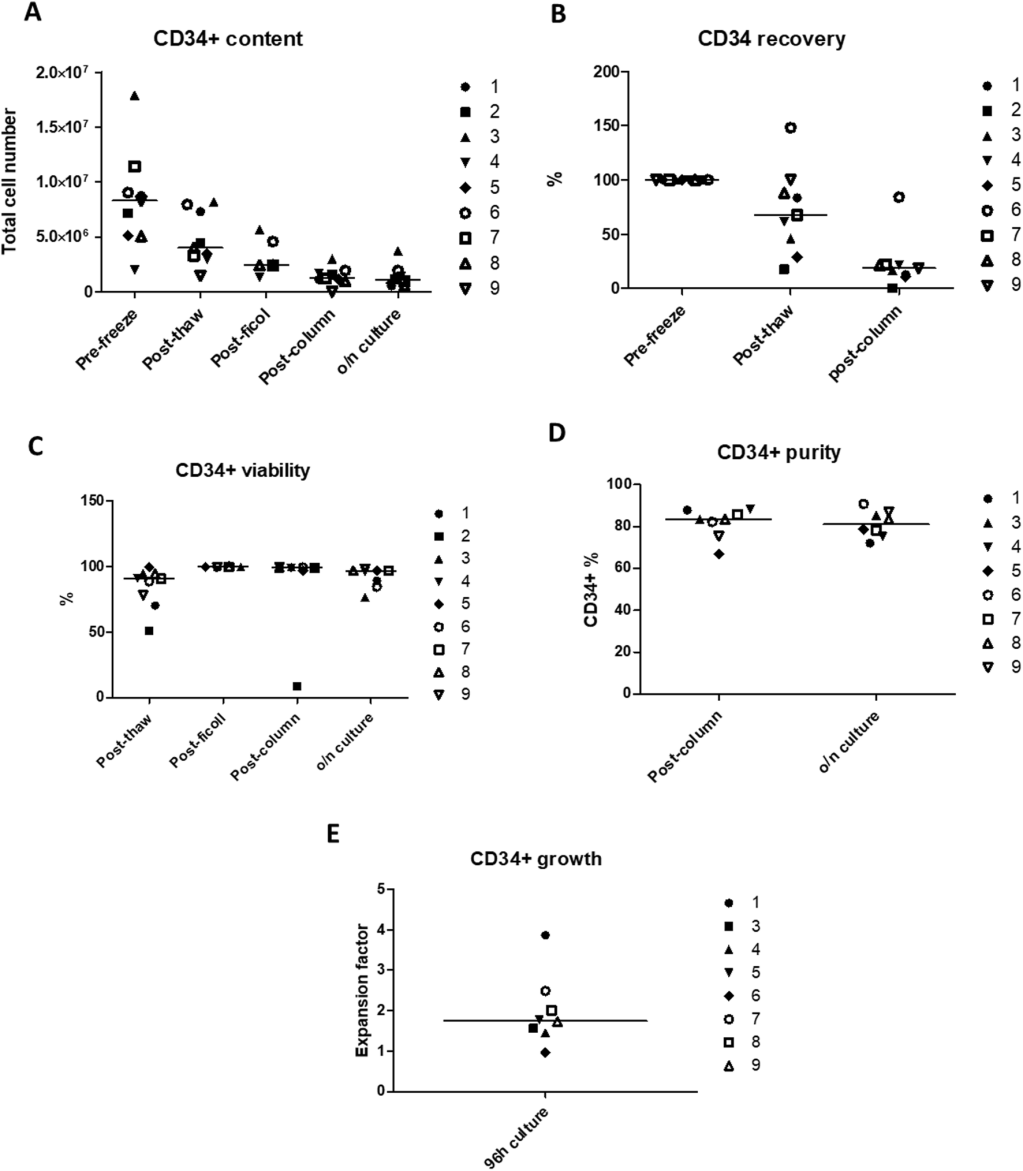
CD34+ population recovery during the process. **A** Total number of CD34+ measured by flow cytometry. **B** Recovery or % vs the initially reported CD34+ content upon collection. **C** CD34+ viability measured by dye exclusion at the cytometer, at different stages of the process. **D** Purity calculated as % of viable CD34+ within the CD45+ viable population of the immunoselected cells just after the purification column and after o/n incubation. **E** Purified CD34+ population expansion after 96 h in culture. Lines depict median values

The range of CD34+ content of the CBU pre-freeze ranged from 2 to 17.9 10^6^ cells and viability was a median 98%. Upon thawing, the median viability was still very high, 90.5% [50.9–99.3%], and the median recovery of CD34+ was 67.6% [17.5–88.1%]. The time of storage in cryopreservation, ranging from 5.1 to 22.8 years, did not seem to affect the viability or the recovery (Fig. 3).

For CD34+ selection we used the clinical grade immunolabelled beads from Miltenyi (CliniMACS CD34 reagent) but not the CliniMACS device, since the device, column and tubing system are designed for larger volumes and much higher content of CD34+ (up to 6 × 10^8^). The CD34+ purification was thus performed in an open system in an A grade hood within a D grade environment. A clinical grade separation column placed in a fixed strong magnetic field was used to select the labelled cells. To improve the purity of the final population, the cells passed through two selection columns sequentially. Figure 3 shows the qualitative analysis during CD34+ isolation. One of the CB units (process n. 2) did not perform to the required standards and the process did not proceed after column purification. We suspect this was due to the quality of the freezing process and not the thawing and isolation, since the quality standards and NC yield were low since the first steps of the process. For the rest of the samples, after column purification we obtained a median of 1.39 × 10^6^ total CD34+ [0.94 × 10^6^–2.98 × 10^6^] cells, resulting in a median recovery of 20% [10.6–84%] of the collected CD34+ and a 34.1% [15.1–56.8%] of the content at thawing. The median viability at this point was 99.1% [96.7–99.5%] and the purity 83.5% [66.9–87.9%]. After column selection the cells were incubated o/n in CD34+ expansion medium and quality tested again. The average total number of CD34+ slightly declined o/n (median 1.05 × 10^6^ CD34+ [0.53 × 10^6^–3.73 × 10^6^]) but viability and purity remain quite constant (medians 96.1% [76.3–98%] and 83.5% [66.9–88.3%], respectively). Samples were taken for sterility testing after o/n incubation and for mycoplasma testing after 72 h in culture, giving a negative result in all cases. In all cases, the purified CD34+ population was allowed to expand for 4 days before reprogramming. After an initial drop in cell number, by day 4 the CD34+ cells had recovered and were proliferating again. The median expansion fold at this point was 1.75 [0.96–3.87]. 1 × 10^4^ CD34+ were used for reprogramming and the rest were kept as a backup and as control for future quality control assays.

## Discussion

Since the number of potential clinical applications for iPSC-derived cells increases and clinical trials progress, the need for clinical grade quality iPSC becomes more evident. The use of banked CB units as a source of HLA homozygous donor cells to create allogenic iPSC cell therapies has become a sensible and attractive option as well as a way to revalorize existing CBB. Contrary to peripheral blood or other tissues as starting material for reprogramming, banked CB does not require new donations and the samples are already screened for medical fitness and tested for viral infections and sterility.

Japan and Korea have reported the production of the first sets of haplolines for their national haplobanks and other countries, such as Germany, Spain or Australia, have initiated the construction of theirs. The IPS-PANIA project aims at the production of seven clinical grade haplolines representing top haplotypes regarding HLA-match coverage of the Spanish population. Not to deploy the Spanish CB registry of any particular HLA haplotype and safeguard the potential for hematopoietic progenitor transplantation, only those haplotypes that were represented in at least two homozygous CB units we considered. This did not affect the top four most common haplotypes but for some of the less common haplotypes we could not find sufficient homozygous CB units.

Since the original CB donation had a specific aim for hematopoietic progenitor transplantation and the particular nature of iPSC cells—unlimited expansion and ever-growing possibilities of applications and potentially multiple patients—the recontacting for the signing of a new Informed Consent document deserves special care. The donors have to be clearly informed of the new nature of the donation and the wide possibilities of the samples. In our experience, contacting the donors by telephone by qualified personnel not directly involved in the project was an efficient way to track back the donors and a favorable predisposition to participate was high. Adapting to the COVID-19 pandemic restrictions and switching from in-person interview to phone calls was well accepted. Most of the recontacted donors (82%) agreed to the new use of the donation.

We set a series of requirements on the homozygous CBU to be selected for consideration, regarding firstly cellularity and CD34+ content, to assure a sufficient number of cells to reprogram and for backup storage after purification and recovery. Although having an O group was initially considered preferential but no an essential requirement, as ABO blood group incompatibility has been described as a possible risk of graft rejection [23], and we found availability of O type donors to for the haplotypes providing the widest coverage, we decided to include it in the requirements list. Similarly, a minimum requirement of homozygosity in HLA-A, HLA-B and HLA-DRB1 was originally set, but preference was given to CBU that were also homozygous for HLA-C. Indeed, all the selected units chosen for reprogramming in the IPS-PNIA project are also homozygous for the HLA-C allele. Rhesus type and sex were not considered as selection requirements as there is no evidence that there could a significant impact in the future engraftment of the iPSC-derived cells.

To thaw and enrich the mononuclear fraction we decided to use the Sepax cell processor, without prior washing, similarly as it was described by Kaur and colleagues [24]. For the CD34+ purification, Liektde and colleagues [19, 21] have reported satisfactory results processing HLA-homozygous CBU for the creation of a haplobank in Germany using a CliniMACs device. Considering the small scale in volume and CD34+ content of cryopreserved CBU we decided not to use the CliniMACS device, which is designed for larger samples and up to tow orders of magnitude higher content of CD34+. We also considered the high cost in reagents and consumables of using the ClinicMACS methods for cryopreserved CB samples. Alternatively, we used a scaled amount of the CliniMACS CD34 reagent and clinical grade columns, and performed a manual immunoselection. Using the described method, we obtained good quality CD34+, with a good recovery, which proliferated and reprogrammed satisfactorily—only one of the eight thawed CBU failed to reprogram.

### Conclusions

We report our own experience in producing HLA-homozygous CD34+ from cryopreserved CBU as staring material for clinical grade iPSC and we propose an efficient and reproducible protocol, from donor recontacting to a cost-effective and clinical grade-compliant CD34+ isolation and expansion procedure. Our aim is to contribute to the production of internationally harmonized haplolines as sources of cell products for clinical research and therapy.

## Supplementary Information

**Additional file 1: Fig. 1.** Health questionnaire carried out on cord blood donors once they agree to participate in the study.

## Data Availability

All the presented data is available for consultation.

## Abbreviations

CB: Cord blood
CBB: Cord blood bank
CBU: Cord blood unit
E-clone: Expected clonogenic efficiency
iPSC: Induced pluripotent stem cells
NC: Nucleated cells
