# Supplementary material for: Public Cord Blood Banks as a source of starting material for clinical grade HLA-homozygous induced pluripotent stem cells
Source: Stem Cell Res Ther. 2022 Aug 12;13:408. doi: 10.1186/s13287-022-02961-6 (PMC9372949; doi:10.1186/s13287-022-02961-6)
Supplement: Supplementary file 1 — Additional file 1: Fig. 1. Health questionnaire carried out on cord blood donors once they agree to participate in the study. [file 13287_2022_2961_MOESM1_ESM.docx]

# HEALTH QUESTIONNAIRE FOR CORD BLOOD DONORS – IPS-PANIA

Name of donor (mother):

Maternity: Cord code:

Date of delivery: Date of questionnaire:

|  | **YES** | **NO** | **Observations** |
| --- | --- | --- | --- |
| **About your son/daughter** |  |  |  |
| How is your son /daughter overall health? |  |  |  |
| Has he/she been diagnosed with any disease? Which one? |  |  |  |
| Has he/she had any blood test done? Why? |  |  |  |
| Has he /she ever been admitted to hospital? Why? |  |  |  |
| Has he/she had any surgery? Which one? |  |  |  |
| **Sobre la mare** |  |  |  |
| 1. **DURING PREGNANCY** |  |  |  |
| 1.1- Did you have any special medication or vaccine? |  |  |  |
| 1.2- Did you visit the dentist? |  |  |  |
| 1.3- Did you had fever or general discomfort? |  |  |  |
| 1.4- Were you in contact with any person suffering an infectious disease or carrying the Hepatitis C virus? |  |  |  |
| 1.5- Were you admitted to hospital or did you have any relevant diagnostic test taken (endoscopy)? |  |  |  |
| 1.6- Did you have any acupuncture treatment, tattoo or piercing? |  |  |  |
| 1.7- Did you have contact with another person's blood from an accidental needle stick? |  |  |  |
| 1.8- Did you travel outside Spain? Where to? |  |  |  |
| 1. **THROUGHOUT YOUR LIVE:** |  |  |  |
| 2.1- Have you ever had any medication for alopecia or skin problems? |  |  |  |
| 2.2- Have you ever had any notable illness? Yes Which one? |  |  |  |
| 2.3- Have you ever had a blood or coagulation factors transfusion? |  |  |  |
| 2.4- Have you received growth hormone of human origin before 1987? |  |  |  |
| 2.5- Have you had any organ or tissue transplant? |  |  |  |
| 1.9- Have you lived outside Spain? Where? (If in the UK, in which years?) |  |  |  |
| 1. **FINAL QUESTIONS** |  |  |  |
| 3.1- Was it your first pregnancy? No How many you had before? |  |  |  |
| 3.2- Are you and the father of the child consanguineous? |  |  |  |
| 3.3- Are you a Blood donor? Yes Have you ever been excluded? |  |  |  |
| **About parents and grandparents** |  |  |  |
| What is the country of origin of the paternal and maternal grandparents? |  |  |  |

**Conclusion:**

Revised by (Name and signature)
